# Association between the Co-administration of Histamine H_2_ Receptor Antagonists and the Effectiveness of Capecitabine in Patients with Colorectal Cancer: Propensity Score Analysis

**DOI:** 10.7150/jca.73385

**Published:** 2022-08-08

**Authors:** Tomoko Yamazaki, Ryuji Uozumi, Hitoshi Kawazoe, Yoshiko Kitazume, Hirotoshi Iihara, Hironori Fujii, Masaya Takahashi, Takahiro Arai, Yasushi Murachi, Yumiko Sato, Takahiro Mikami, Koji Hashiguchi, Tomoe Yoshizawa, Katsuyuki Takahashi, Yukiyoshi Fujita, Yuki Hosokawa, Issei Morozumi, Masami Tsuchiya, Atsushi Yokoyama, Hironobu Hashimoto, Tetsuya Furukawa

**Affiliations:** 1 Department of Pharmacy, Tochigi Cancer Center, 4-9-13 Yohnan, Utsunomiya, Tochigi 320-0834, Japan.; 2 Department of Biomedical Statistics and Bioinformatics, Kyoto University Graduate School of Medicine, 54 Kawahara-cho, Shogoin, Sakyo-ku, Kyoto 606-8507, Japan.; 3 Division of Pharmaceutical Care Sciences, Center for Social Pharmacy and Pharmaceutical Care Sciences, Keio University Faculty of Pharmacy, 1-5-30 Shibakoen, Minato-ku, Tokyo 105-8512, Japan.; 4 Division of Pharmaceutical Care Sciences, Keio University Graduate School of Pharmaceutical Sciences, 1-5-30 Shibakoen, Minato-ku, Tokyo 105-8512, Japan.; 5 Department of Pharmacy, National Cancer Center Hospital, 5-1-1 Tsukiji, Chuo-ku, Tokyo 104-0045, Japan.; 6 Department of Pharmacy, Gifu University Hospital, 1-1 Yanagido, Gifu, Gifu 501-1194, Japan.; 7 Department of Pharmacy, Osaka City University Hospital, 1-5-7 Asahi-machi, Abeno-ku, Osaka 545-8586, Japan.; 8 Division of Pharmacy, Gunma Prefectural Cancer Center, 617-1 Takahayashi-nishi-cho, Ota, Gunma 373-0828, Japan.; 9 Department of Pharmacy, Independent Administrative Institution Higashiosaka City Medical Center, 3-4-5 Nishiiwata, Higashiosaka, Osaka 578-8588, Japan.; 10 Department of Frontier Science for Cancer and Chemotherapy, Osaka University Graduate School of Medicine, 2-2 Yamadaoka, Suita, Osaka 565-0871, Japan.; 11 Department of Pharmacy, Nagoya City University West Medical Center, 1-1-1 Hirate-cho, Kita-ku, Nagoya, Aichi 462-8508, Japan.; 12 Department of Pharmacy, Miyagi Cancer Center, 47-1 Nodayama, Medeshimashiote, Natori, Miyagi 981-1293, Japan.; 13 Department of Pharmacy, Yokohama Minami Kyousai Hospital, 1-21-1 Mutsuurahigashi, Kanazawa-ku, Yokohama, Kanagawa 236-0037, Japan.

**Keywords:** capecitabine, CapeOX, histamine H_2_ receptor antagonist, drug-drug interaction, colorectal cancer

## Abstract

**Background:** The association between the effectiveness of capecitabine and the concomitant administration of gastric acid suppressants remains controversial. We aimed to clarify whether the effectiveness of capecitabine is affected by the co-administration of histamine H_2_ receptor antagonists (H_2_RAs) in early-stage colorectal cancer (CRC) patients using real-world data.

**Methods:** This multicenter, retrospective, observational study included consecutive patients with stage II-III CRC who received either capecitabine monotherapy or the CapeOX regimen (capecitabine and oxaliplatin) as adjuvant therapy between January 2009 and December 2014 in Japan. Relapse-free survival (RFS) and overall survival were estimated using the Kaplan-Meier method. Additionally, multivariable Cox proportional hazards model, propensity score adjustment, and inverse probability of treatment weighting analyses were performed.

**Results:** In total, 552 patients were included in this study, of which 30 were co-administered H_2_RAs. RFS at five years was 76.7% (95% confidence interval [CI]: 57.2-88.1%) and 79.8% (95% CI: 76.0-83.0%) in the H_2_RA and non-H_2_RA groups, respectively. Multivariable Cox proportional hazards model and propensity score-adjusted analyses showed that the co-administration of H_2_RAs was associated with a poor RFS among those receiving capecitabine monotherapy (hazard ratio [HR], 2.01; 95% CI: 0.86-4.70 and HR, 1.81; 95% CI: 0.77-4.22, respectively). In contrast, these results were inconsistent with the group receiving the CapeOX regimen.

**Conclusions:** The study findings suggest that the co-administration of H_2_RAs may not reduce the effectiveness of capecitabine therapy in patients with early-stage CRC. To confirm this relationship, a prospective study with a pharmacokinetic approach is needed.

## Introduction

According to a leading global cancer statistics source, colorectal cancer (CRC) was the third most commonly diagnosed cancer (10.0%) and the second leading cause of cancer death (9.4%) in both the sexes in the year 2020 1. Capecitabine is an oral prodrug designed for supplying high concentrations of 5-fluorouracil in tumor cells. It is commonly used for treating solid tumors, including CRC, gastric, as well as breast cancer, according to the package insert in Japan. Several recent studies have suggested that the concomitant use of proton pump inhibitors (PPIs) and capecitabine reduces the effectiveness of capecitabine in CRC 2-5. Our previous study investigated the clinical consequences of the concomitant administration of PPIs and capecitabine monotherapy or the CapeOX regimen (capecitabine and oxaliplatin) in patients with early-stage CRC, where we found that the co-administration of PPIs led to poor survival outcomes 6. PPIs are used to manage peptic ulcers and gastroesophageal reflux disease, and are among the most widely prescribed drugs among patients with cancer 7, 8. Previous studies indicate that drug-drug interactions (DDIs), reduction of capecitabine solubility, or an increased CRC risk associated with PPIs may explain the association between PPI co-administration and the reduced effectiveness of capecitabine. However, the underlying mechanism remains unclear 9-12, which precludes the possibility of a therapeutic strategy that can effectively maintain the effectiveness of capecitabine.

Histamine H_2_ receptor antagonists (H_2_RAs) are acid-suppressive medications similar to PPIs 7. In Japan, H_2_RAs are widely prescribed for gastrointestinal disorders. However, there are a few reports on combining capecitabine with H_2_RAs in CRC patients 4, 12. Additionally, the clinical impact of the co-administration of H_2_RAs with capecitabine as postoperative adjuvant treatment in early-stage CRC patients has not been evaluated. Other studies have suggested that the carcinogenic risk of the long-term administration of H_2_RAs is nil or lower than that related to the administration of PPIs 13-15.

The purpose of this study was to clarify whether the co-administration of H_2_RAs affects the effectiveness of capecitabine monotherapy and CapeOX regimen in early-stage CRC patients using real-world data.

## Methods

### Patients

This was a multicenter, retrospective, observational study, and was conducted at nine institutions in Japan. Data were collected from the medical records of each institution, and compiled at the National Cancer Center Hospital; subsequently, data analyses were performed at the Keio University Faculty of Pharmacy and Kyoto University Graduate School of Medicine. The manuscript was prepared with reference to the STROBE checklist 16.

The inclusion criteria were as follows: 1) consecutive patients aged ≥ 20 years with pathologically diagnosed stage II-III CRC and who had undergone curative surgery; and 2) patients who had received at least one course of adjuvant capecitabine monotherapy (2,500 mg/m^2^, days 1-14, every 3 weeks) or the CapeOX regimen (capecitabine 2,000 mg/m^2^ on days 1-14, plus oxaliplatin 130 mg/m^2^ on day 1, every 3 weeks) between January 2009 and December 2014. The clinicopathological findings were used for reclassification according to the TNM Classification of Malignant Tumors 8^th^ edition, published by the Union for International Cancer Control 17. The treatment schedule and follow-up duration were modified at the clinician's discretion according to the toxicity profile of each patient.

The exclusion criteria were as follows: 1) refused use of medical records; 2) insufficient or missing information in the medical records; 3) history of the administration of capecitabine monotherapy or CapeOX regimen prior to the investigation period; 4) prior administration of any adjuvant chemotherapy, except for capecitabine monotherapy or the CapeOX regimen; 5) prior administration of any neoadjuvant chemotherapy; 6) concurrent radiotherapy during adjuvant chemotherapy; 7) inadequate bone marrow, liver, and renal function at baseline (neutrophil count < 1,500 cells/mm^3^ or white blood cell count < 3,000 cells/mm^3^; hemoglobin < 9.0 g/dL; platelet count < 75,000 cells/mm^3^; total bilirubin > 2.25 mg/dL; aspartate transaminase > 60 U/L, alanine transaminase > 84 U/L for men and > 46 U/L for women, and creatinine clearance ≤ 51 mL/min as calculated by the Cockcroft-Gault equation); 8) more than eight cycles of adjuvant chemotherapy; 9) development of other carcinomas after receiving adjuvant chemotherapy; and 10) co-administration of PPIs which was defined as a ≥ 20% overlap between PPI administration and adjuvant chemotherapy administration 3.

### Data collection

Patient records were de-identified and analyzed anonymously. The following data were collected: age, sex, body surface area, cancer stage, TNM classification of malignant tumors, primary tumor site, chemotherapy regimen and dose, concomitant PPI or H_2_RA use, laboratory data before chemotherapy, and date of recurrence and/or death. The primary site included the right-sided colon (defined as the cecum, ascending colon, and transverse colon), left-sided colon (defined as the descending colon, sigmoid, and rectosigmoid junction), and rectum. The relative dose intensity (RDI) of the capecitabine or CapeOX regimens was defined as the percentage of actual dose intensity per scheduled dose intensity of eight courses. Concomitant use of H_2_RAs was defined as a ≥ 20% overlap between H_2_RA administration and capecitabine administration in accordance with a previous study 3. The follow-up period ended on December 31, 2019.

### Endpoints

Relapse-free survival (RFS) was defined as the period from the date of capecitabine administration to the date of radiographic recurrence or death from any cause. Overall survival (OS) was defined as the period from the date of capecitabine administration to the date of death from any cause. Patients who were still alive as well as those without documented radiographic recurrence were censored at the date of the last follow-up. The primary and secondary endpoints were RFS and OS, respectively.

### Statistical analysis

RFS and OS in the H_2_RA and non-H_2_RA groups were estimated using the Kaplan-Meier method; confidence intervals (CIs) were calculated using the complementary log-log transformation and Greenwood's formula. The follow-up period was analyzed using reverse Kaplan-Meier estimates 18. Subsequently, a multivariable Cox proportional hazards model was applied to compare the differences between the two groups. Hazard ratios (HRs) and 95% CIs were presented. Potential explanatory variables concerning patient background including chemotherapy regimen (CapeOX vs. capecitabine), concomitant use of H_2_RAs (yes vs. no), age (10-year intervals), sex (male vs. female), primary site (right-sided colon vs. others (left-sided colon and/or rectal)), cancer stage (III high-risk (T4, N2, or both cancers) vs. III low risk (T1, T2, or T3, and N1 cancers) vs. II), and RDI (10% intervals) were included as covariates in the multivariable model 19-21. To account for indication bias due to the lack of randomization, propensity score-adjusted analyses were performed using the following: 1) a multivariable model including the propensity score as an additional covariate, and 2) an inverse probability of treatment weighting (IPTW) method 22, 23. The propensity score of H_2_RA co-administration was estimated for each patient using a logistic regression model 24. According to the recommendation of the American Statistical Association 25, 26, a *P* < 0.05 should be avoided when interpreting *P*-values; therefore, we interpreted the results on the basis of point estimates with their CIs. Furthermore, to supplement conventional CIs, we performed a *post-hoc* analysis using the Cox model re-expressed in a Bayesian statistical framework. We computed the Bayesian posterior probability 27 of HR < 1 based on a non-informative prior distribution as a reference to evaluate the hypotheses concerning the direction and magnitude of the unknown HR via the Cox model. We did not impute any missing data. All statistical analyses were performed using SAS version 9.4 and JMP version 16.2.0 (SAS Institute, Cary, NC, USA).

### Ethics statement

Ethical approval was provided by the National Cancer Center Institutional Review Board (Approval No. 2019-294), ethics committee of the Tochigi Cancer Center (Approval No. 20-A001), medical review board of Gifu University Graduate School of Medicine (Approval No. 2020-069), ethical committee of Osaka City University Graduate School of Medicine (Approval No. 2020-042), ethics committee of the Gunma Prefectural Cancer Center (Approval No. 405-02012), ethical review board of Osaka University Hospital (Approval No. 20008), Nagoya City University East/West Medical Center Institutional Review Board (Approval No. 20-04-423-03), ethics review committee of the Miyagi Cancer Center (Approval No. 2020-003), and the ethics committee of the Yokohama Minami Kyosai Hospital (Approval No. 1-20-4-1) in Japan. This study was conducted in accordance with the Declaration of Helsinki and the Ethical Guidelines for Medical and Health Research involving Human Subjects promulgated by the Ministry of Education, Culture, Sports, Science, and Technology and the Ministry of Health, Labour, and Welfare of Japan. Acquiring written or oral informed consent from participants was waived considering the retrospective nature of the study. Therefore, we used an opt-out method through the official website of each participating institution.

## Results

### Patient characteristics

**Figure 1:**
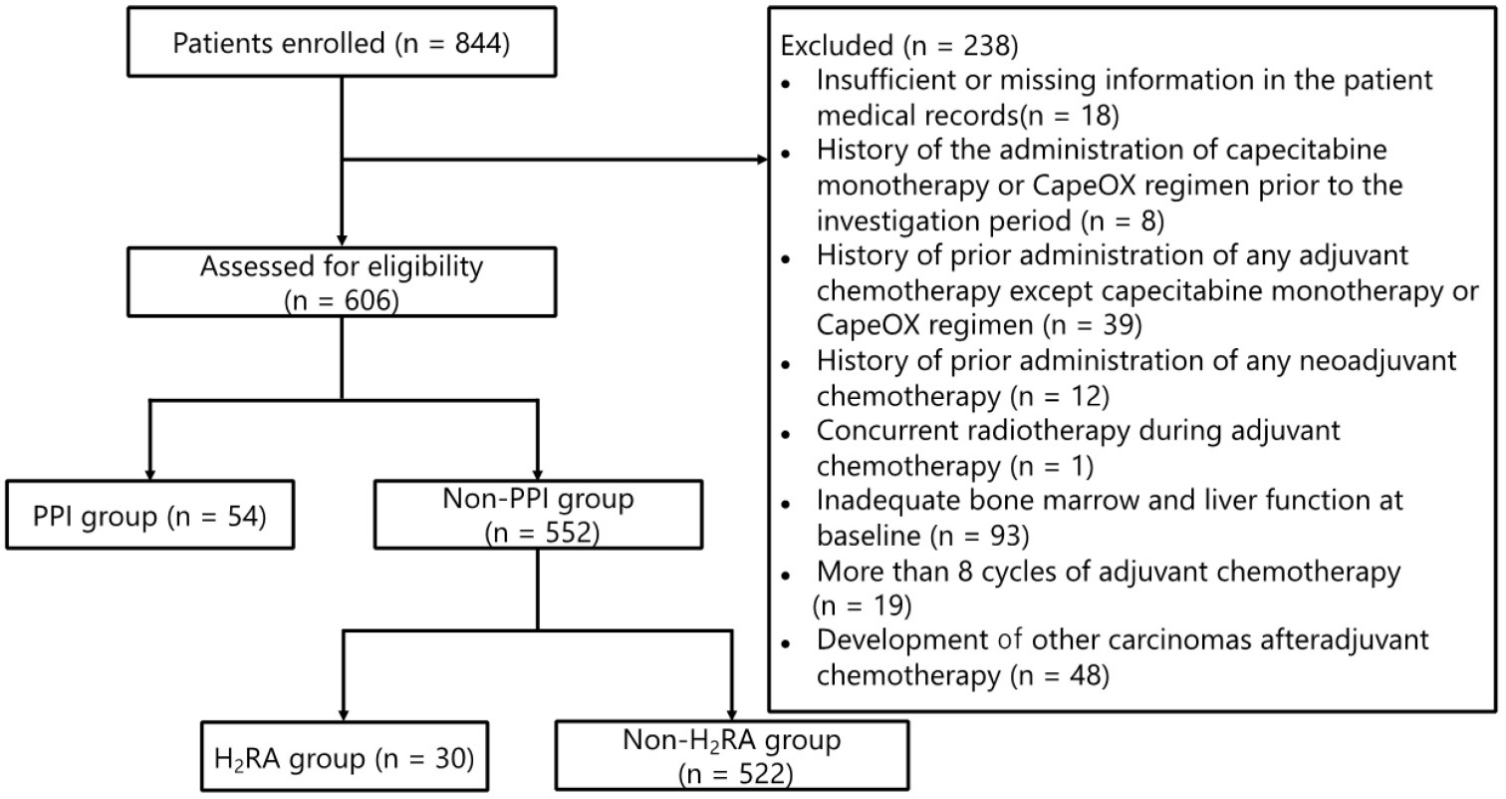
** Patient enrollment flowchart.** Abbreviations: CapeOX: capecitabine and oxaliplatin; PPI: proton pump inhibitor; H_2_RA: Histamine H_2_ receptor antagonist

The patient flowchart illustrating the enrollment process is shown in **Figure 1**. Of the 844 patients who were initially screened, 238 were withdrawn from the analysis on the basis of the exclusion criteria as detailed in the Methods section. Subsequently, 54 patients who had received PPIs were further excluded from the analysis. Thus, data pertaining to 552 patients were evaluated in this study, of which 30 (5.4%) received H_2_RAs; of these 30 patients, 20 (66.7%) and 10 (33.3%) received capecitabine monotherapy and the CapeOX regimen, respectively.

The baseline patient characteristics are listed in **Table 1**. The median age of the patients was 63 years (interquartile range (IQR): 55-70 years), of which 305 (55.3%) were men, and 161 (29.2%) had right-sided colon cancer. In the H_2_RA group, the median duration of concomitant H_2_RA use was 100.0% (IQR: 87.5%-100%).

### Endpoints

The median duration of follow-up was 6.1 years (95% CI: 5.9-6.3 years). Overall, there were 110 relapse events and 66 deaths. Among patients who received capecitabine monotherapy (2,500 mg/m^2^, days 1-14, every 3 weeks, 8 cycle), the median RDI of capecitabine was 81.1% (IQR: 64.2-87.8%) and 79.3% (IQR: 65.0-91.3%) in the H_2_RA and non-H_2_RA groups, respectively. Among patients who received the CapeOX regimen (capecitabine at 2,000 mg/m^2^ on days 1-14, plus oxaliplatin at 130 mg/m^2^ on day 1, every 3 weeks, 8 cycles), the median RDI of capecitabine was 84.9% (IQR: 79.1-91.1%) and 75.1% (IQR: 62.5-87.4%) in the H_2_RA and non-H_2_RA groups, respectively, and the median RDI of oxaliplatin was 72.4% (IQR: 59.6-81.8%) and 66.3% (IQR: 45.6-78.9%) in the H_2_RA and non-H_2_RA groups, respectively.

**Figure 2:**
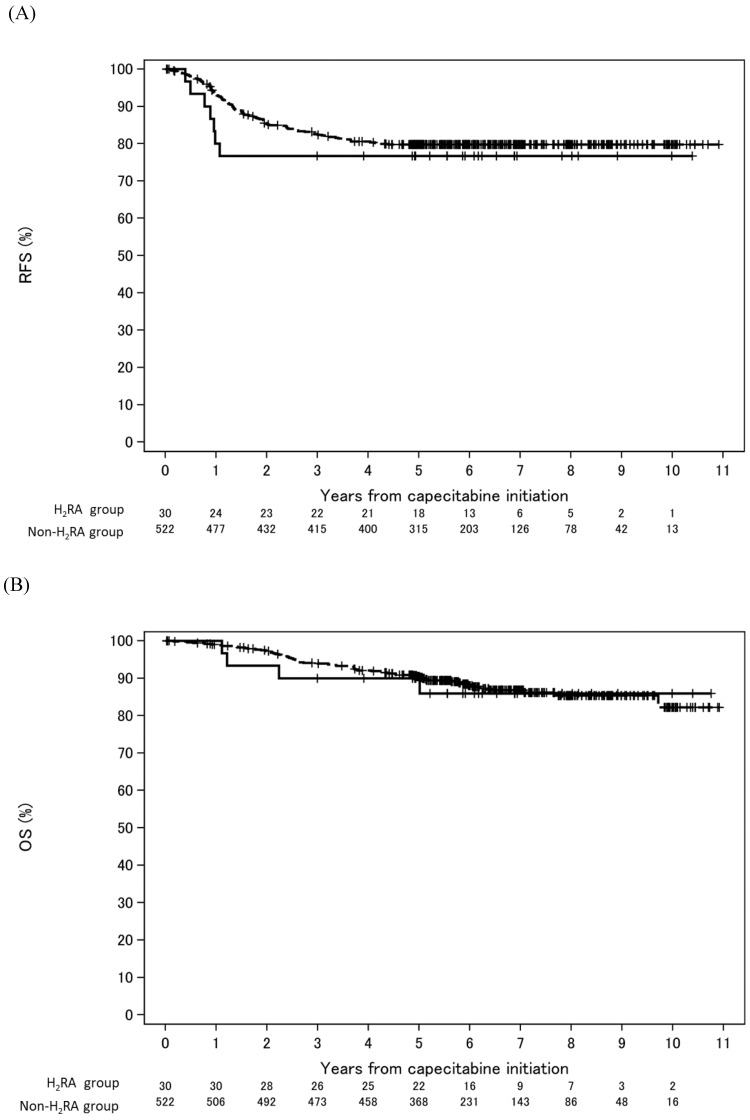
** Kaplan-Meier survival curves estimate according to the absence or presence of H_2_RA co-administration.** The solid and dashed lines are H_2_RA group and non-H_2_RA group, respectively. (A) Relapse-free survival**.** (B) Overall survival**.** Abbreviations: H_2_RA: histamine H_2_ receptor antagonist; RFS: relapse-free survival; OS: overall survival

As shown in **Figure 2**, in the entire study population (capecitabine monotherapy and CapeOX regimen), the RFS at five years was 76.7% (95% CI: 57.2-88.1%) and 79.8% (95% CI: 76.0-83.0%) in the H_2_RA and non-H_2_RA groups, respectively. The OS at five years was 90.0% (95% CI: 72.1-96.7%) and 90.4% (95% CI: 87.5-92.7%) in the H_2_RA and non-H_2_RA groups, respectively. According to the univariable analysis, there were no significant differences in RFS and OS between the H_2_RA and non-H_2_RA groups (RFS: HR, 1.28; 95% CI: 0.59-2.74; *P* = 0.533 and OS: HR, 1.09; 95% CI: 0.40-3.00; *P* = 0.867, respectively).

As shown in **Table 2**, the multivariable Cox proportional hazards model and propensity score-adjusted analyses revealed that the co-administration of H_2_RAs was associated with shortened RFS to a small degree (HR, 1.12; 95% CI: 0.52-2.42, *P* = 0.772 and HR, 1.18; 95% CI: 0.55-2.53, *P* = 0.677, respectively). In contrast, OS was inconsistent as compared with RFS (**Table 3**).

A comparison between the capecitabine monotherapy and the CapeOX regimen groups is shown in **Tables 4 and 5**. The multivariable Cox proportional hazards model and propensity score-adjusted analyses showed that the co-administration of H_2_RAs was relatively associated with a relatively poor RFS in the capecitabine monotherapy group (HR, 2.01; 95% CI: 0.86-4.70; *P* = 0.108 and HR, 1.81; 95% CI: 0.77-4.22; *P* = 0.172, respectively), although no significant difference was observed. In contrast, inconsistent results were obtained for the CapeOX regimen group with respect to RFS.

In the capecitabine monotherapy population, the Bayesian posterior probability showed that the HRs for the RFS of the H_2_RA group relative to that of the non-H_2_RA group would be < 1.00, ranging from 8.4% to 47.0% (**Table 4**). In the CapeOX regimen population, the Bayesian posterior probability showed that the HRs for the RFS of the H_2_RA group relative to that of the non-H_2_RA group would be < 1.00, ranging from 87.0% to 96.6% (**Table 5**).

## Discussion

In the present study, we found that in real-world clinical practice, the effectiveness of capecitabine monotherapy and CapeOX regimen is unlikely to be affected by the combination of H_2_RAs in early-stage CRC patients. However, multivariable Cox proportional hazards model and propensity score-adjusted analyses showed that the co-administration of H_2_RAs was associated with a poor RFS among those receiving capecitabine monotherapy (HR, 2.01; 95% CI: 0.86-4.70 and HR, 1.81; 95% CI: 0.77-4.22, respectively). The HR of RFS was higher for capecitabine monotherapy and tended to fall in the overall population (capecitabine monotherapy and CapeOX). The difference in HR may have been attributed to the increased intensity of treatment with the addition of oxaliplatin and the different dosage of capecitabine in the two treatment regimens.

To date, few studies have examined the influence of H_2_RAs on the effectiveness of capecitabine therapy. Rhinehart et al. 4 observed that the co-administration of antacids (PPIs and H_2_RAs) affects the effectiveness of capecitabine, but the number of H_2_RAs users in that study was small in two cases. In a similar study, Kichenadasse et al. 12 reported no association between H_2_RA co-administration and worse OS and PFS (n = 362). The results were obtained from six randomized clinical trials, but patients with early-stage CRC were not included. In both the above-mentioned studies, the difference in RDI between the H_2_RA and non-H_2_RA groups was unclear, which may have affected the results. Notably, the present study evaluated the RDI of capecitabine; the difference in the RDI of capecitabine between the H_2_RA and non-H_2_RAr groups was only 1.8% in the capecitabine monotherapy-treated population (higher in the H_2_RA user group). Cancer patients may be prescribed H_2_RAs to reduce gastrointestinal symptoms; however, it was not clear whether this led to an increase in the RDI. The other known risk factors for recurrence after adjuvant chemotherapy in CRC include tumor invasiveness (T) and lymph node status (N) 28, 29. To ascertain the impact of these factors, sensitivity analyses were performed with concomitant H_2_RA (yes vs. no), age (10-year interval), sex (male vs. female), primary site (right-sided colon vs. other), chemotherapy regimen (CapeOX vs. capecitabine), and cancer stage (III high-risk (T4, N2, or both cancers) vs. III low risk (T1, T2, or T3 and N1 cancers) vs. II) as covariates. The number of H_2_RA-treated patients in this study was relatively small (n = 30), but the overall study population was large (n = 552); the study included several cancer centers, university hospitals, and community hospitals. To the best of our knowledge, this is the first study to clarify whether the co-administration of H_2_RAs affects the effectiveness of capecitabine therapy in patients with early-stage CRC in a real-world setting, and therefore our results have considerable clinical implications.

Previous studies have reported that the co-administration of PPIs may have a negative impact on the effectiveness of capecitabine in patients with early-stage or advanced CRC and gastroesophageal cancer 2-6. The results of this study suggest that although PPIs and H_2_RA are antacids, their impact on capecitabine therapy differs. The therapeutic effect of capecitabine therapy may be maintained by replacing PPIs with H_2_RAs. To determine whether this is possible, it is necessary to clarify the mechanism by which the PPIs combination reduces the effect of capecitabine. This evidence suggests that acid-reducing agents (ARAs) may reduce the effectiveness of capecitabine treatment. Several hypotheses have been proposed regarding the mechanism by which PPIs attenuate the effect of capecitabine. One hypothesis states that a DDI between the PPIs and capecitabine leads to reduced capecitabine efficacy. According to this hypothesis, capecitabine is sensitive to changes in pH, and a PPI-induced increase in gastric pH reduces the absorption of capecitabine. In previous studies, ARAs have been shown to affect the effectiveness of oral anticancer agents 30. However, according to a systematic review of DDIs pertaining to ARAs 31, elevated gastric pH is a common characteristic of all three classes of ARAs (antacids, H_2_RAs, and PPIs). Therefore, clinically important gastric pH-mediated interactions should be observed with all ARAs, and if PPIs reduce the effect of capecitabine due to changes in pH, then H_2_RAs should have the same result. Several *in vivo* studies have examined the influence of ARAs on the pharmacokinetics of capecitabine. *In vivo* studies on DDIs between capecitabine and ARAs have reported no interaction between capecitabine and Maalox^®^ (dried aluminum hydroxide gel, magnesium hydroxide) in 12 patients with solid tumors 32, 33, and no interaction between capecitabine and rabeprazole in patients with CRC 11. Accordingly, PPIs may be less likely to show gastric pH-dependent interactions with capecitabine. Another hypothesis suggests that the use of PPIs itself may affect CRC. It has been reported that the suppression of gastric acidity by PPIs and H_2_RAs led to hypergastrinemia and induced the proliferation of colorectal epithelium and progression of colonic adenoma in *in vivo* models 34-36. Additionally, while several case-control studies have concluded that PPI use was associated with an increased risk of CRC 13, one cohort study reported no increase in CRC risk 37. The above studies agree that H_2_RA administration is not a risk factor for CRC. However, an increased risk of gastric cancer was reported with H_2_RA use 38. Thus, risk evaluation pertaining to these drug classes must be performed and clarified by future studies. It is necessary to confirm that the concentrations of capecitabine and its metabolites are adequately high in patients receiving concomitant PPIs and H_2_RAs, and examine the *in vivo* pharmacokinetics of these drugs and evaluate differences among these and other drugs that are used to treat the same indication. The effect of the timing of the dose administration on DDIs should also be clarified. It is possible that multiple mechanisms are involved in the PPIs-induced reduction of capecitabine efficacy, and comorbidities may play a role as well. Therefore, prospective studies are needed to explore the mechanism underlying the reduction of capecitabine efficacy by PPIs. While the mechanism underlying this phenomenon could not be clarified in this study.

The present study has some limitations. First, it was a retrospective, observational study rather than a prospective study. The present study evaluated patients according to the guidelines of the Japanese Society for Cancer of Colon and Rectum (JSCCR); these guidelines are used during the treatment of CRC in actual clinical practice in Japan. Therefore, we excluded 93 patients whose major organ functions were not preserved at the time of therapy initiation. The JSCCR guidelines were published in 2009, 2010, and 2014 39, 40. The new additions in the JSCCR guidelines 2014 state that postoperative chemotherapy should be started 4-8 weeks after surgery, and that the CapeOX regimen has to be covered by insurance. As this is a retrospective study, information bias cannot be ruled out. Thus, multivariable analysis was performed to reduce the effect of potential confounding factors that were related to patient characteristics. Second, the sample sizes of the H_2_RA and non-H_2_RA groups were not equal. Notably, the number of H_2_RA-treated patients was relatively small, due to which it might not have been evaluated satisfactorily. We were unable to evaluate the OS in the population that received the CapeOX regimen due to the small number of events. Furthermore, there was a large variability regarding the Bayesian posterior probability shown in Tables 2 (range: 0.388-0.607) and 4 (range: 0.084-0.461), which might be an overestimation of IPTW owing to the small number of H_2_RA-treated patients. Third, the H_2_RA data were based on prescription information from the medical records; therefore, information on whether the patients purchased and used an over-the-counter drug was not available. Furthermore, it was unclear whether the patients took H_2_RAs during capecitabine treatment and whether medication adherence was adequate. Forth, the baseline laboratory data prior to chemotherapy represented the latest value in this study, and was therefore unable to identify the exact day within a given number of days. Fifth, we did not collect baseline CEA level or comorbidity data and therefore were not included as covariates in the multivariable model; the primary reasons for not collecting these data are as follows: First, information regarding comorbidity was not available in every institution because of the retrospective nature of the study. Second, patients with severe complications did not undergo surgery or postoperative adjuvant chemotherapy; hence, we focused on RFS, which included death due to any reason, rather than cancer-specific recurrence and death. Sixth, the frequency and timing of diagnostic imaging varies between facilities and may therefore affect the diagnosis of recurrence, which is a potentially confounding factor. Future prospective studies with larger sample sizes are necessary to confirm the study findings, evaluate the pharmacokinetic aspects of the drugs, and explore the mechanism underlying the effect of ARAs on the effectiveness of capecitabine therapy.

## Conclusions

The findings of this study suggest that the concomitant use of H_2_RAs in patients with early-stage CRC receiving capecitabine therapy is unaffected by an increased risk of recurrence. Our data provide preliminary evidence for an association between the co-administration of H_2_RAs and capecitabine efficacy in Japanese patients with stage II-III CRC.

## Figures and Tables

**Table 1:** Baseline patient characteristics

Characteristic		All (n = 552)	H_2_RAgroup (n = 30)^a^		Non-H_2_RA group (n = 522)^a^	
			Capecitabine monotherapy (n = 20)	CapeOX (n = 10)	Capecitabine monotherapy (n = 400)	CapeOX (n = 122)
Age, median (IQR), y		63 (55-70)	68 (60-74)	59 (53-63)	64 (57-71)	60 (50-67)
Sex						
	Male	305 (55.3)	12 (60.0)	6 (60.0)	214 (53.5)	73 (59.8)
	Female	247 (44.7)	8 (40.0)	4 (40.0)	186 (46.5)	49 (40.2)
Primary site^a^						
	Right-sided colon	161 (29.2)	2 (10.0)	6 (60.0)	121 (30.3)	32 (26.2)
	Left-sided colon	200 (36.2)	12 (60.0)	2 (20.0)	149 (37.3)	37 (30.3)
	Rectum	191 (34.6)	6 (30.0)	2 (20.0)	130 (32.5)	53 (43.4)
Stage						
	II	66 (12.0)	1 (5.0)	1 (10.0)	59 (14.8)	5 (4.1)
	IIIA	96 (17.4)	4 (20.0)	1 (10.0)	77 (19.3)	14 (11.5)
	IIIB	315 (57.1)	12 (60.0)	6 (60.0)	225 (56.3)	72 (59.0)
	IIIC	75 (13.6)	3 (15.0)	2 (20.0)	39 (10.0)	31 (25.4)
Co-administered H_2_RA						
	Famotidine		15 (75.0)	7 (70.0)		
	Ranitidine		5 (25.0)	0 ( 0.0)		
	Lafutidine		0 ( 0.0)	3 (30.0)		

Abbreviations: CapeOX: capecitabine and oxaliplatin; IQR: interquartile range; H_2_RA: histamine H_2_ receptor antagonist; y: years. ^a^ Percentages may not add up to 100 because of rounding.

**Table 2:** Multivariable Cox proportional hazards model, propensity score-adjustment, and IPTW analyses of the effect of the co-administration of H_2_RA on RFS with capecitabine monotherapy and the CapeOX regimen

					Multivariable analysis			Adjusted for propensity score			IPTW		
Variables		No.	Event	Censored	HR (95% CI)	*P*	Posterior probability	HR (95% CI)	*P*	Posterior probability	HR (95% CI)	*P*	Posterior probability
H_2_RA	Yes	30	7	23	1.12 (0.52-2.42)	0.772	0.435	1.18 (0.55-2.53)	0.677	0.391	0.76 (0.32-1.80)	0.527	0.776
	No	522	103	419	1			1			1		
Age (10-year intervals)		-	-	-	0.88 (0.74-1.04)	0.130							
Sex	Male	305	69	236	1.43 (0.97-2.11)	0.071							
	Female	247	41	206	1								
Primary site	Right-sided colon	161	34	127	1.05 (0.69-1.58)	0.831							
	Others	391	76	315	1								
Stage	III high-risk	179	59	120	2.15 (1.15-4.00)	0.016							
	III low-risk	307	39	268	0.70 (0.37-1.35)	0.289							
	II	66	12	54	1								

Abbreviations: H_2_RA: histamine H_2_ receptor antagonist; CapeOX: capecitabine and oxaliplatin; CI: confidence interval; HR: hazard ratio; IPTW: inverse probability of treatment weighting; RFS: relapse-free survival.

**Table 3:** Multivariable Cox proportional hazards model, propensity score-adjustment, and IPTW analyses of the effect of the co-administration of H_2_RA on OS with capecitabine monotherapy and the CapeOX regimen

					Multivariable analysis			Adjusted for propensity score			IPTW		
Variables		No.	Event	Censored	HR (95% CI)	*P*	Posterior probability	HR (95% CI)	*P*	Posterior probability	HR (95% CI)	*P*	Posterior probability
H_2_RA	Yes	30	4	26	0.82 (0.30-2.27)	0.704	0.699	0.90 (0.32-2.49)	0.836	0.644	0.66 (0.22-1.96)	0.458	0.816
	No	522	62	460	1			1			1		
Age (10-year intervals)		-	-	-	0.92 (0.73-1.15)	0.450							
Sex	Male	305	44	261	1.74 (1.04-2.91)	0.035							
	Female	247	22	225	1								
Primary site	Right-sided colon	161	24	137	1.33 (0.79-2.21)	0.281							
	Others	391	42	349	1								
Stage	III high-risk	179	42	137	2.80 (1.19-6.61)	0.018							
	III low-risk	307	18	289	0.65 (0.26-1.64)	0.362							
	II	66	6	60	1								

Abbreviations: H_2_RA: histamine H_2_ receptor antagonist; CapeOX: capecitabine and oxaliplatin; CI: confidence interval; HR: hazard ratio; IPTW: inverse probability of treatment weighting; OS: overall survival.

**Table 4:** Multivariable Cox proportional hazards model, propensity score-adjustment, and IPTW analyses of the effect of the co-administration of H_2_RA on RFS with capecitabine monotherapy

					Multivariable analysis			Adjusted for propensity score			IPTW		
Variables		No.	Event	Censored	HR (95% CI)	*P*	Posterior probability	HR (95% CI)	*P*	Posterior probability	HR (95% CI)	*P*	Posterior probability
H_2_RA	Yes	20	6	14	2.01 (0.86-4.70)	0.108	0.084	1.81 (0.77-4.22)	0.172	0.126	1.12 (0.31-4.04)	0.864	0.470
	No	400	71	329	1			1			1		
Age (10-year intervals)		-	-	-	0.85 (0.69-1.04)	0.115							
Sex	Male	226	47	179	1.45 (0.92-2.31)	0.113							
	Female	194	30	164	1								
Primary site	Right-sided colon	123	24	99	1.13 (0.68-1.87)	0.631							
	Others	297	53	244	1								
Stage	III high-risk	110	35	75	2.28 (1.12-4.63)	0.023							
	III low-risk	250	32	218	0.77 (0.38-1.57)	0.477							
	II	60	10	50	1								

Abbreviations: H_2_RA: histamine H_2_ receptor antagonist; CI: confidence interval; HR: hazard ratio; IPTW: inverse probability of treatment weighting; RFS: relapse-free survival.

**Table 5:** Multivariable Cox proportional hazards model, propensity score-adjustment, and IPTW analyses of the effect of the co-administration of H_2_RA on RFS with the CapeOX regimen

					Multivariable analysis			Adjusted for propensity score			IPTW		
Variables		No.	Event	Censored	HR (95% CI)	*P*	Posterior probability	HR (95% CI)	*P*	Posterior probability	HR (95% CI)	*P*	Posterior probability
H_2_RA	Yes	10	1	9	0.27 (0.03-2.02)	0.200	0.962	0.27 (0.04-2.05)	0.206	0.966	0.52 (0.07-4.20)	0.543	0.870
	No	122	32	90	1			1			1		
Age (10-year intervals)		-	-	-	0.95 (0.69-1.31)	0.740	-						
Sex	Male	79	22	57	1.33 (0.64-2.74)	0.446							
	Female	53	11	42	1								
Primary site	Right-sided colon	38	10	28	1.14 (0.53-2.46)	0.736							
	Others	94	23	71	1								
Stage	III high-risk	69	24	45	0.98 (0.22-4.35)	0.979							
	III low-risk	57	7	50	0.29 (0.06-1.47)	0.136							
	II	6	2	4	1								

Abbreviations: H_2_RA: histamine H_2_ receptor antagonist; CI: confidence interval; CapeOX: capecitabine and oxaliplatin; HR: hazard ratio; IPTW: inverse probability of treatment weighting; RFS: relapse-free survival.

## Abbreviations

ARAs: acid-reducing agents
CapeOX: capecitabine and oxaliplatin
CI: confidence interval
CRC: colorectal cancer
DDIs: drug-drug interactions
H_2_RAs: Histamine H_2_ receptor antagonists
HR: hazard ratio
IPTW: inverse probability of treatment weighting
IQR: interquartile range
JSCCR: Japanese Society for Cancer of Colon and Rectum
OS: overall survival
PPIs: proton pump inhibitors
RDI: relative dose intensity
RFS: relapse-free survival
